# Store-operated Ca^2+^ entry regulates glioma cell migration and invasion via modulation of Pyk2 phosphorylation

**DOI:** 10.1186/s13046-014-0098-1

**Published:** 2014-11-30

**Authors:** Meng Zhu, Lei Chen, Pengfei Zhao, Hua Zhou, Chen Zhang, Shengping Yu, Yu Lin, Xuejun Yang

**Affiliations:** Department of Neurosurgery, Tianjin Medical University General Hospital, 154# Anshan Road, Tianjin, 300052 China; Laboratory of Neuro-Oncology, Tianjin Neurological Institute, Tianjin, 300052 China

**Keywords:** Store-operated Ca^2+^ entry, Glioma, Focal adhesion turnover, Epithelial-to-mesenchymal (−like) transition, Proline-rich tyrosine kinase 2

## Abstract

**Background:**

The ubiquitous second messenger Ca^2+^ has been demonstrated to play an important role in cancer progression. Store-operated Ca^2+^ entry (SOCE) is the main Ca^2+^ entry pathway regulating intracellular Ca^2+^ concentration in a variety of cancer types. The present study aimed to explore the specific mechanisms of SOCE in the processes of glioma migration and invasion.

**Methods:**

The expression of Orai1, a key component of SOCE, was examined in glioma samples and glioma cell lines by immunohistochemistry and western blot analysis. Both pharmacological intervention and RNA interference were employed to investigate the role of SOCE in glioma cell migration and invasion in vitro. The intracellular Ca^2+^ was certified through Fluo-4/AM based Ca^2+^ measurement. The effect of SOCE on cell viability, migration, and invasion was explored by methyl thiazolyl tetrazolium (MTT) assay, wound healing assay, transwell invasion assay. Western blot analysis and immunofluorescence assay were used to observe the changes of downstream related protein and cell morpholog.

**Results:**

Orai1 expression was elevated in glioma tissues and several glioma cell lines compared with non-neoplastic brain tissues. Either inhibition of SOCE by a pharmacological inhibitor or Orai1 downregulation suppressed glioma cell migration and invasion. However, re-expression of Orai1 could rescue glioma cell motility. Furthermore, phosphorylation of proline-rich tyrosine kinase 2 (Pyk2) participated in the mechanisms by which SOCE regulated focal adhesion turnover and epithelial-to-mesenchymal (−like) transition in glioma cells, both of which are considered to be critical for tumor progression.

**Conclusions:**

The SOCE-Pyk2 pathway is essential for glioma migration and invasion. The study indicates the potential value of Orai1 as a molecular target for anti-invasion therapy.

**Electronic supplementary material:**

The online version of this article (doi:10.1186/s13046-014-0098-1) contains supplementary material, which is available to authorized users.

## Background

Gliomas are the most common primary tumors in the central nervous system (CNS), and glioblastoma multiforme (GBM) has the poorest prognosis among glioma types. Even with the current optimal therapeutic strategies, GBM patients have a median survival of only 12–15 months after diagnosis [1]. Clinical and histologic evidence has shown that glioma cells always disperse along thin and elongated anatomic structures such as white matter fibers, capillaries, and unmyelinated axons [2]. For this reason, glioma cells cannot be completely resected by surgical treatment, which leads to recurrence and poor prognosis. Therefore, new treatment approaches that inhibit glioma cell invasion and migration represent as urgent medical need. The identification of new molecular regulators related to tumor progression may provide potential targets for future therapeutic strategies.

The ubiquitous intracellular second messenger Ca^2+^ plays an important role in many fundamental physiological processes, including cell excitability, exocytosis, motility, apoptosis, and transcription [3]. Recent research indicates that Ca^2+^ also contributes to several malignant behaviors in tumors, such as proliferation, invasion, migration, and metastasis [4,5]. There are a variety of Ca^2+^ entry pathways in cells. Store-operated Ca^2+^ entry (SOCE), which is initiated by the depletion of intracellular Ca^2+^ stores, is an important pathway in nonexcitable cells [6]. SOCE is mediated by store-operated Ca^2+^ channels (SOCs), including stromal interacting molecule-1 (STIM1) and Orai1. The vast majority of STIM1 is located in the endoplasmic reticulum (ER) membrane, and Orai1 is located in the plasmalemma. When external stimuli cause Ca^2+^ release from the ER, store depletion is sensed by STIM1. STIM1 then moves near to the cell membrane and interacts directly with Orai1. As the essential pore-forming component of SOCs, Orai1 opens and mediates entry of many Ca^2+^ ions. Recently, SOCE has been implicated in tumor cell progression. Inhibition of SOCE was shown to suppress human breast cancer cell migration both in vitro and in vivo [7]. The specific mechanisms include SOCE-mediated induction of a higher rate of focal adhesion turnover and accelerated migration velocity of cancer cells, whereas a reduction in SOCE resulted in larger focal adhesions, slowing their turnover and consequently increasing adherence. Similar studies were performed in cervical cancer and hepatocarcinoma, and the results also support the above conclusion [8,9].

One study of SOCE in glioblastoma found suppression of SOCE inhibits human glioblastoma cell proliferation and induces G0/G1 phase arrest [10]. Another research group found that downregulation of STIM1 and Orai1 in primary human glioblastoma cell lines results in a significant decrease in tumor cell invasion in vitro [11]. However, the study did not investigate the morphological changes of tumor cells and the specific downstream mechanisms. In the current study, we verified the expression of Orai1 in different grades of glioma tissues and several glioma cell lines. More importantly, we found that SOCE regulates focal adhesion turnover and epithelial-to-mesenchymal (−like) transition (EMT-like) in glioma cells by modulating proline-rich tyrosine kinase 2 (Pyk2) phosphorylation.

## Methods

### Cell culture

The human glioma cell lines U251, SNB19, U87, and LN229 and the rat glioma cell line C6 were purchased from the Chinese Academy of Sciences Cell Bank (Beijing, China). All cell lines were cultured in Dulbecco’s Modified Eagle’s Medium (DMEM) supplemented with 10% fetal bovine serum (FBS) (Solarbio, Beijing, China) in an atmosphere of 5% CO_2_ at 37°C.

### Sample collection

Glioma samples were obtained from 61 patients by surgical resection in the Department of Neurosurgery, Tianjin Medical University General Hospital between July 2008 and December 2012. Eight non-neoplastic normal brain tissues were obtained from patients with temporal lobe epilepsy. For immunohistochemical analysis, samples were fixed in 4% paraformaldehyde and embedded in paraffin. Samples for western blot analysis were stored in liquid nitrogen. The pathological diagnosis and grading for each glioma were assessed by neuropathologists according to the 2007 World Health Organization (WHO) Classification of Nervous System Tumors [12]. All samples were obtained at primary resection, including 13 low-grade glioma samples (WHO II, n = 13) and 48 high-grade glioma samples (WHO III, n = 12; WHO IV, n = 36), and none of the patients had undergone radiation therapy or chemotherapy before surgery. All patients and their relatives provided written informed consent. Sample collection was performed in accordance with the ethical standards of the Helsinki Declaration and approved by the ethical committed of Tianjin Medical University General Hospital.

### Antibodies and reagents

The following antibodies were used: rabbit monoclonal anti-Orai1 and mouse monoclonal anti-vinculin (Abcam, Cambridge, UK); rabbit polyclonal anti-Pyk2 and mouse monoclonal anti-E-cadherin (Santa Cruz Biotechnology, Santa Cruz, CA, USA); mouse monoclonal anti-phosphorylated Pyk2 (p-Pyk2) (Tyr402; R&D Systems, Minneapolis, MN, USA); rabbit monoclonal anti-N-cadherin and rabbit monoclonal anti-vimentin (Cell Signaling Technology, Danvers, MA, USA); Alexa Fluor 594-conjugated goat anti-mouse IgG (H + L) antibody (Invitrogen, Carlsbad, CA, USA); Alexa Fluor 488-conjugated goat anti-rabbit IgG (H + L) antibody (Cell Signaling Technology).

Important reagents were as follows: thapsigargin, SKF96365, puromycin, and G418 from Sigma-Aldrich (St. Louis, MO, USA); and Fluo-4/AM and Pluronic-127 from Invitrogen. Boyden chambers were purchased from Millipore (Billerica, MA, USA) and Matrigel was purchased from BD Biosciences (San Jose, CA, USA). Confocal Petri dishes were obtained from NEST Biotechnology (Wuxi, JS, China).

### Immunohistochemistry

Paraffin-embedded samples were sectioned using a microtome into 5-μm-thick sections for immunohistochemical staining. Nonspecific proteins were blocked using goat serum, and then the slides were incubated separately in the primary antibody solution (rabbit anti-Orai1, 1:200 dilution) overnight at 4°C. Antibodies bound to Orai1 were stained with DAB substrate after conjugation using the horseradish peroxidase-conjugated secondary antibody. Images were acquired using an Olympus VANOX microscopy at magnifications of × 100 and × 200. The results were evaluated by two independent pathologists. The intensity of positively stained cells was scored from 0–3 according to the extent of staining from 0%–100%: 0 for 0%, 1 for 1–33%, 2 for 34–66%, and 3 for 67–100%.

### Western blot analysis

Western blot analysis was carried out as previously described [13]. Samples were broken into small pieces, and cells were cultured to 90% confluence before harvesting. After total proteins were extracted, 30 μg of each sample was analyzed by sodium dodecyl sulfate (SDS)-polyacrylamide gel electrophoresis (PAGE) on 10% acrylamide gels and processed using the antibodies listed above. Western blot analysis was performed with an enhanced chemiluminescence (ECL) kit (Millipore). Each experiment was repeated three times independently. Quantitative evaluation of protein expression was performed using ImageJ software (National Institutes of Health, Bethesda, MD, USA). The average gray values of target proteins (normalized to that for glyceraldehyde 3-phosphate dehydrogenase GAPDH expression) are presented in the figures.

### Cell viability assay

The methyl thiazolyl tetrazolium (MTT) assay was used to evaluate cell viability. Cells were plated in a 96-well culture plate (5 × 10^3^ cells/well) in regular growth medium for 24 h. Then cells were treated with 20 μM SKF96365 and maintained in culture for 72 h. At time points of 0, 24, 48, and 72 h, assays were initiated by adding 20 μl MTT substrate to each well and incubating cells for another 4 h to allow metabolism of the MTT. Finally, the medium was removed and 200 μl dimethyl sulfoxide (DMSO) was added to each well. The absorbance of each well was read at 490 nm using an automated microplate reader (Bio-Rad, Hercules, CA, Canada). All experiments were performed in triplicate.

### Wound healing assay

Glioma cells were evenly plated in a 6-well culture plate and allowed to reach 70% confluence. Then wounds were made by scratching the cell layer using a 200-μl sterile pipette tip. In the presence of serum, cells should migrate and fill the wound within approximately 48 h. Images were acquired using an Olympus IX71 inverted microscope at magnification of × 100. The images shown are representative of three independent experiments. The numbers of migrated cells between the two edges of the gap in five random fields were counted for further quantitative analysis.

### Transwell invasion assay

Boyden chambers with a pore size of 8 μm were coated with Matrigel in DMEM (1:3 ratio) in advance. Then Boyden chambers were coated with 20 μl of the compound evenly and incubated at 37°C for 30 min. Glioma cells (5 × 10^4^ in 200 μl DMEM without FBS) were plated on the top side of the Matrigel-coated Boyden chambers. The lower compartments were filled with DMEM supplemented with 10% FBS. After incubation for 36 h, the non-invasive cells on the upper surface of the membranes were gently removed using cotton swabs and the invasive cells on the lower surface were fixed with 4% paraformaldehyde, stained with crystal violet, and counted (five random fields per well). All experiments were repeated three times independently.

### Immunofluorescence assay

Cells were plated in confocal Petri dishes coated with poly-L-lysine (1 mg/ml) and incubated overnight for adherence. Then, cells were fixed at room temperature with 4% paraformaldehyde for 10 min and permeabilized with 0.1% Triton X-100. Cells were incubated with primary antibodies overnight at 4°C and stained by Texas Red or fluorescein isothiocyanate (FITC)-labeled secondary antibody. Cell nuclei were counterstained with 4′,6-diamidino-2-phenylindole (DAPI) staining solution. All images were taken using an Olympus FV-1000 confocal microscope.

### RNA interference and rescue experiment

Small hairpin RNA (shRNA) directed against Orai1 was generated using the GV112 vector (U6-MCS-CMV-puromycin) (GeneChem, Shanghai, China). The sequence used was 5′-CGTGCACAATCTCAACTCG-3′ [7]. Cells transfected with shOrai1 were selected using puromycin (5 μg/ml). The cDNA construct for re-expression of Orai1 was obtained by site-directed mutation of the targeting sequences without changing the amino acid sequence. The mutant was subcloned into a GV141 vector (CMV-MCS-3FLAG-SV40-Neomycin) (GeneChem, Shanghai, China). Cell stably transfected with shOrai1 were transfected with the Orai1 rescue construct and selected again using G418 (1 μg/ml). The shRNA plasmid for Pyk2 knockdown was purchased from Santa Cruz Biotechnology and was a pool of three target-specific 19–25-nt small interfering RNAs (siRNAs) designed to knock down gene expression. The control shRNA plasmid was also provided. Lipofectamine 2000 reagent (Invitrogen) was used for transfection of the Orai1 construct and shPyk2. The whole process was performed according to the manufacturer’s instructions.

### Intracellular Ca^2+^ measurement

To measure intracellular Ca^2+^ in glioma cells, we used Fluo-4/AM, a cell-permeable fluorescent Ca^2+^ indicator, as previously described [14]. The standard solution for Ca^2+^ measurement contained 140 mM NaCl, 2 mM CaCl_2_, 5 mM KCl, 0.45 mM KH_2_PO_4_ ,0.4 mM Na_2_HPO_4_, 1.2 mM MgSO_4_, 1.2 mM MgCl_2_, 4.2 mM NaHCO_3_, 10 mM glucose, and 5 mM HEPES (pH 7.4). Fluo-4/AM was mixed with an equal volume of 20% Pluronic-127 and diluted in standard solution to a final concentration of 5 μM. Glioma cells plated in confocal Petri dishes were washed with standard solution and incubated with Fluo-4/AM for 30 min at 37°C protected from light. Fluo-4–loaded cells were then washed three times and allowed to stabilize for 10 min in standard solution. Cells were then stabilized in Ca^2+^-free solution (which contained 0 Ca^2+^) for 10 min, and again with standard solution for another 10 min. The ER Ca^2+^ ATPase inhibitor thapsigargin (5 μM) was added 3 min after the start of superfusion with Ca^2+^-free solution and was continuously present thereafter. Thapsigargin was used to induce store depletion, which would lead to the activation of SOCE. SOCE activity was checked by measuring the increase in intracellular Ca^2+^ upon return to standard solution. The dynamic change in fluorescence intensity was monitored at 5-s intervals using an Olympus FV-1000 confocal microscope. Data curves were drawn to reflect the dynamic change in intracellular Ca^2+^.

### Statistical analysis

All quantified data represent an average of at least triplicate experiments unless otherwise indicated, and standard deviations were calculated. All statistical analyses were performed using SPSS 19.0 (SPSS, Inc., Chicago, IL, USA) and GraphPad Prism 5.0 (GraphPad Software, La Jolla, CA, USA). Comparisons among all groups were performed using one-way analysis of variance (ANOVA) or unpaired Student’s t-tests. *P* < 0.05 was considered to be statistically significant.

## Results

### Orai1 expression in glioma samples and glioma cell lines

**Figure 1:**
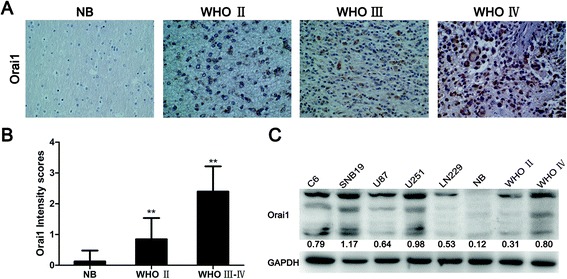
**Expression of Orai1 in glioma samples and glioma cell lines. (A)** Representative patterns of Orai1 expression in non-neoplastic brain tissues and glioma samples of different grades by immunohistochemistry. NB, non-neoplastic brain tissues. Magnification, ×200. **(B)** Intensity scores for STIM1 and Orai1 expression by immunohistochemistry. The data are shown as mean ± standard deviation (SD). **P < 0.01 (WHO II vs. NB, WHO III–IV vs. NB, and WHO III–IV vs. WHO II). **(C)** Western blot analysis of Orai1 expression in non-neoplastic brain tissues, glioma samples (WHO II and IV), and five typical glioma cell lines. The gray values represent the means of three independent experiments (normalized to GAPDH expression).

As one important Ca^2+^ membrane channel protein, Orai1 is the key component that mediates SOCE. To study the role of SOCE in glioma migration and invasion, we first assessed the expression levels and subcellular localization of Orai1 in 61 glioma samples and 8 non-neoplastic brain tissues by immunohistochemistry. Representative immunohistochemical staining patterns for Orai1 are shown in Figure 1A. Positive staining for Orai1 was mainly localized in the cytoplasm and plasmalemma at varying levels. The data showed that non-neoplastic brain tissues expressed low levels of Orai1, but Orai1 expression was positively correlated with the WHO grading of gliomas. Compared with non-neoplastic brain tissues, low-grade gliomas (WHO II, n = 13) displayed slightly elevated Orai1 protein expression, whereas high-grade gliomas (WHO III, n = 12; WHO IV, n = 36) showed a significantly elevated Orai1 expression compared with non-neoplastic brain tissues and low-grade samples (Figure 1B).

Next, Orai1 protein expression was detected in non-neoplastic brain tissues, human glioma samples (WHO II and IV), and five typical glioma cell lines by western blot analysis. The results are shown in Figure 1C. Orai1 was commonly overexpressed at higher levels in glioma samples (WHO II and IV) and in all glioma cell lines compared with non-neoplastic brain tissues.

### SKF96365 inhibited migration and invasion of glioma cells

The overexpression of Orai1 in glioma tissues and cell lines leads us to believe that SOCE may be involved in the migration and invasion of glioma cells. U251 and SNB19 glioma cells were used in the following experiments. First, we investigated the influence of SKF96365 on the malignant behavior in glioma cells. SKF96365, an imidazole compound, was introduced as an pharmacological inhibitor of SOCE in cell lines in the concentration range of 20–30 μM [15]. SKF96365 has been widely used in various recent studies related to SOCE [7,9].

**Figure 2:**
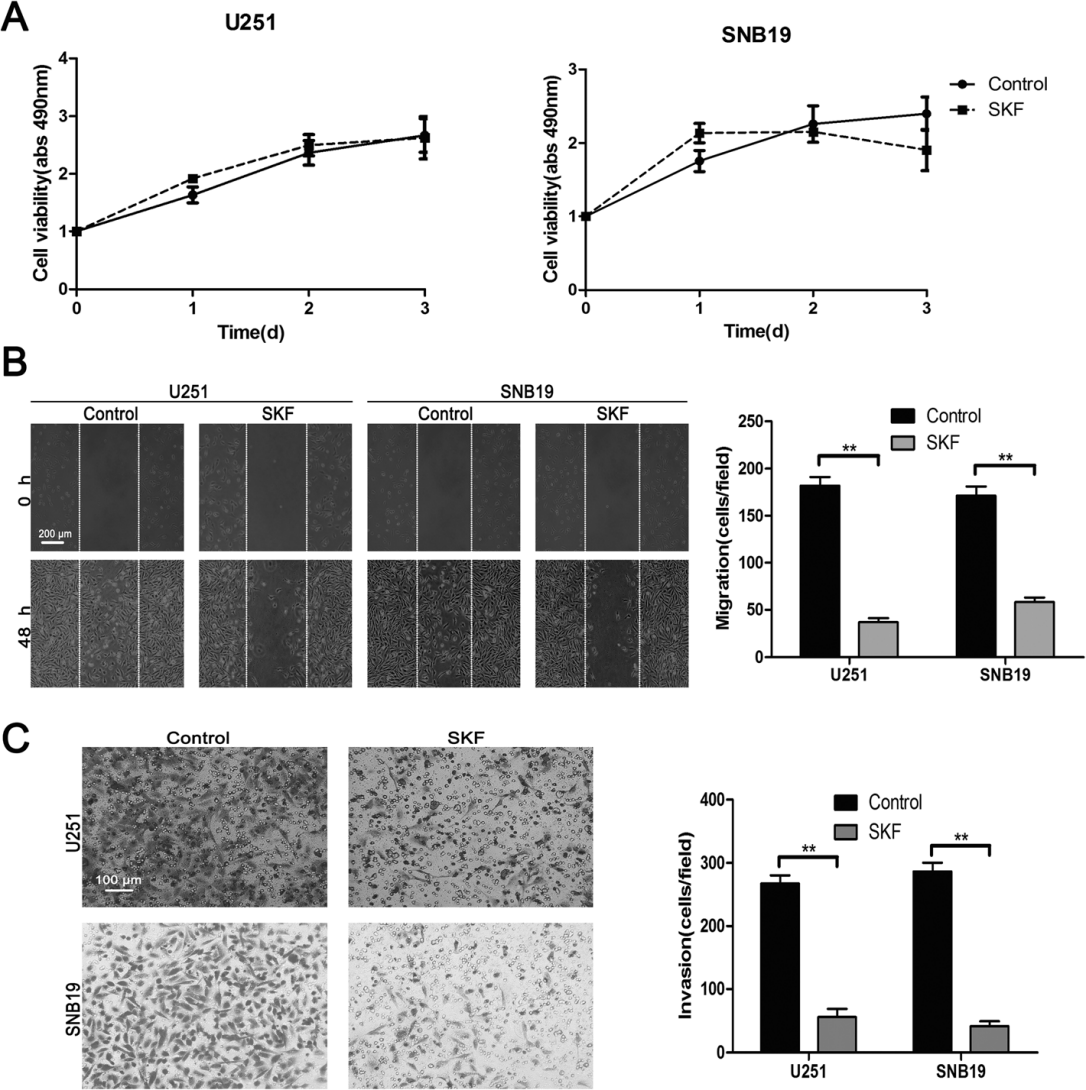
**SKF96365 inhibited migration and invasion of U251 and SNB19 glioma cells. (A)** The effect of SKF96365 at the tested concentration (20 μM) on U251 and SNB19 cell viability as measured by MTT assay. Absorbance was read at 490 nm with averages from triplicate wells. **(B and C)** Wound healing assays and migration assays employing Matrigel-coated Boyden chambers were performed to investigate the effect of SKF96365 on the migration and invasion of U251 and SNB19 cells. Data are presented as means ± SDs of nine different microscopic visions from three independent experiments. **P < 0.01.

We performed MTT assays to evaluate the toxicity of SKF96365 at the tested concentration (20 μM). Following the manufacturer’s instructions, SKF96365 was dissolved in sterile deionized water, which has no effect on glioma cells. We found that the viability of U251/SNB19 glioma cells was not impaired (*P* > 0.05) by treatment with SKF96365 (20 μM) for 72 h compared with the solvent control (Figure 2A).

Wound healing assays were performed to observe the influence of SKF96365 on glioma cell migration. For both U251 and SNB19 cells, SKF96365 significantly inhibited the migration of tumor cells by 66%–80% (Figure 2B). The invasive ability of glioma cells was assessed via migration assays using Matrigel-coated Boyden chambers. The numbers of migrating cells in the SKF96365-treated groups were decreased by 79%–85% compared with the control groups (Figure 2C). These results suggest that SOCE may play an important role in controlling the motility of glioma cells.

### SKF96365 inhibited focal adhesion turnover, EMT-like, and the activity of Pyk2 in glioma cells

Several previous studies have shown that SOCE is involved in tumor cell migration and invasion via modulation of focal adhesion turnover. We therefore sought to determine whether SOCE plays the same role in glioma cells. Therefore, we investigated the expression of several EMT-related markers and the activity of Pyk2, an intracellular calcium-dependent tyrosine kinase that is activated through phosphorylation and considered essential for both focal adhesion disassembly and EMT.

**Figure 3:**
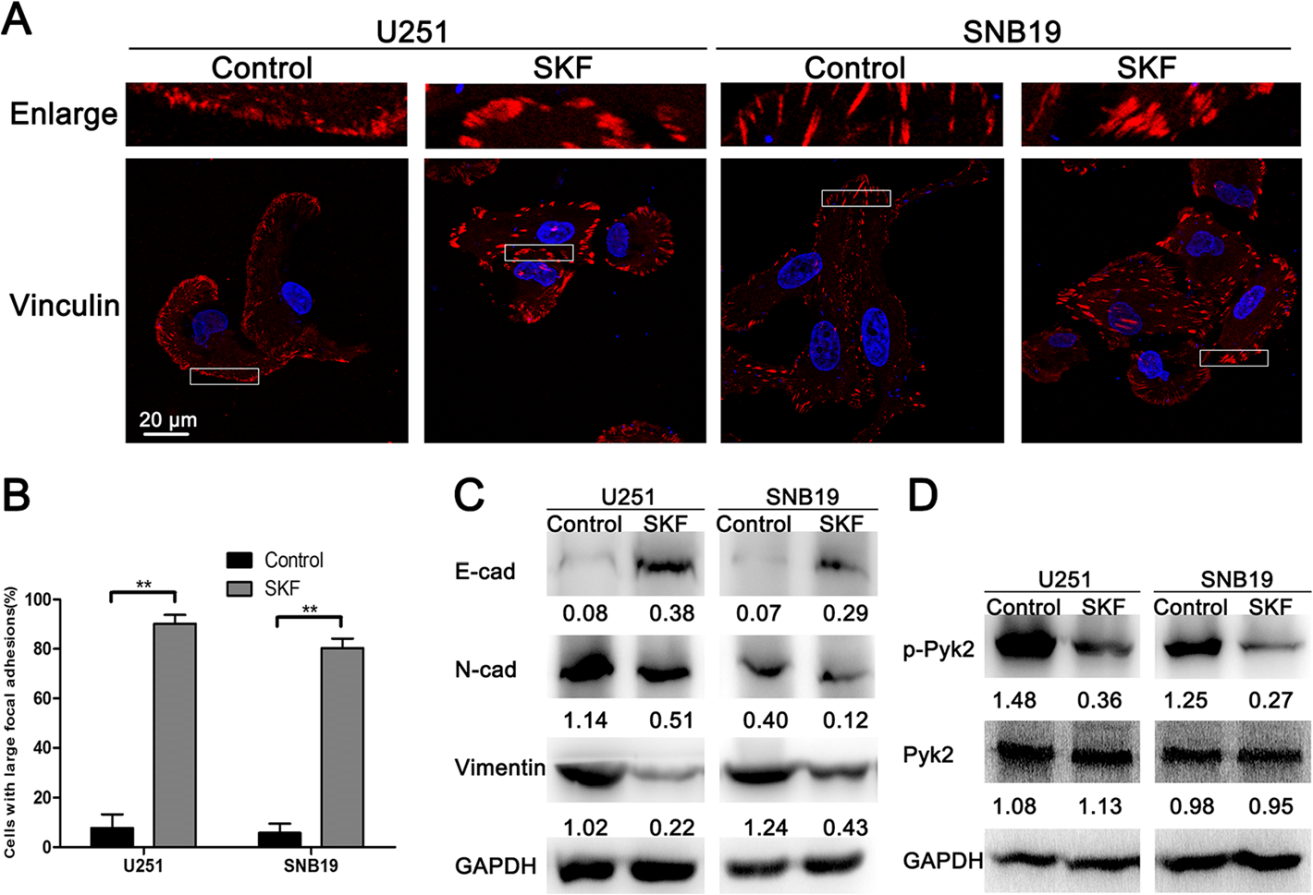
**SKF96365 inhibited focal adhesion turnover, EMT-like, and Pyk2 phosphorylation in U251 and SNB19 glioma cells. (A and B)** Immunofluorescent staining for vinculin showed differences in focal adhesions between the control and SKF96365-treated glioma cells. Magnification, ×1000. The proportions of cells with large focal adhesions are presented as means ± SDs of nine different microscopic visions from three independent experiments. **P < 0.01. **(C)** Western blot analysis of E-cadherin, N-cadherin, and vimentin in U251 and SNB19 cells treated with SKF96365. **(D)** Western blot analysis of the total Pyk2 and p-Pyk2 in U251 and SNB19 cells after treatment with SKF96365. The gray values represent the means of three independent experiments (normalized to GAPDH expression).

Focal adhesions can be visualized by immunofluorescent labeling for vinculin, a major component of focal adhesions. U251/SNB19 cells plated on poly-L-lysine (1 mg/ml)-coated confocal Petri dishes were treated with medium with or without SKF96365 (20 μM) for 3 h, fixed in 4% paraformaldehyde, and then stained with anti-vinculin antibody. Control tumor cells showed a normal morphology characterized by irregular shape and stretched pseudopodia. Vinculin immunofluorescent labeling showed a punctate or threadlike pattern of small focal adhesions in control cells (Figure 3A). However, treatment with SKF96365 led to cell rounding and induced large peripheral adhesions while decreasing the number of adhesions in the center area of the cell (Figure 3A). Large peripheral focal adhesions were observed in 80%–90% of cells treated with SKF96365, but in only 6%–8% of control cells (Figure 3B).

Then, the protein expression of three important EMT-related markers was analyzed by western blotting. The results showed that E-cadherin (an epithelial marker) was expressed at extremely low levels in both glioma cell lines, whereas it was significantly upregulated after treatment with SKF96365 for 3 h (Figure 3C), suggesting a shift towards epithelial phenotype. Consistently, the expression of two mesenchymal markers, N-cadherin and vimentin, was decreased upon treatment with SKF96365 (Figure 3C). Our results clearly demonstrate that treatment with SKF96365 promotes epithelial cell characteristics and suppresses mesenchymal features in glioma cells. Next, we detected the expression and phosphorylation of Pyk2 at the Tyr402 site. The results indicated that the total Pyk2 levels were nearly unchanged, whereas the levels of p-Pyk2 were significantly decreased in both cell lines after treatment with SKF96365 (Figure 3D).

### Orai1 controlled glioma cell motility and phosphorylation of Pyk2

**Figure 4:**
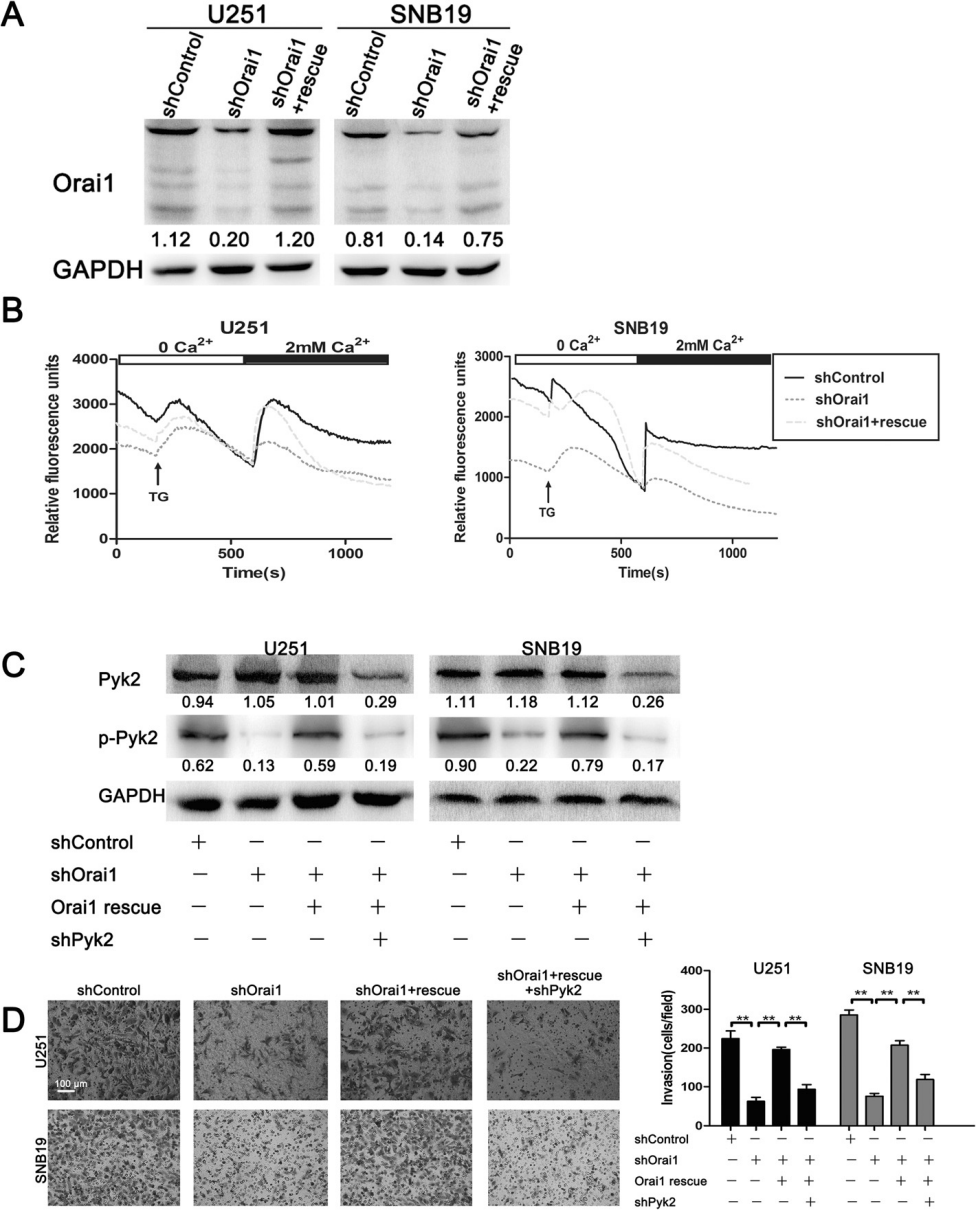
**Orai1 controlled U251 and SNB19 cell invasion through regulation of Pyk2 activity. (A)** The expression of Orai1 in U251 and SNB19 cells transfected with shControl, shOrai1, or Orai1 rescue by western blot analysis. **(B)** Changes in Ca^2+^ influx in U251 and SNB19 cells transfected with shControl, shOrai1, or Orai1 rescue according to Fluo-4 Ca^2+^ measurements. Each trace was obtained by averaging data from 25–45 single cells. TG, thapsigargin. **(C)** Western blot analysis of the total Pyk2 and p-Pyk2 in U251 and SNB19 cells transfected with shControl, shOrai1, Orai1 rescue, or shPyk2. **(D)** Invasive ability of U251 and SNB19 cells transfected with shControl, shOrai1, Orai1 rescue, or shPyk2 as examined by Matrigel-coated Boyden chamber assays. Data are presented as means ± SDs of nine different microscopic visions from three independent experiments. **P < 0.01.

Based on the results presented above, we hypothesized that modulating SOCE via targeting of Orai1 could also impact the motility of glioma cells. Orai1 was stably knocked down in U251/SNB19 cells by transfection with Orai1 shRNA. Western blot analysis and Ca^2+^ measurements were employed to confirm the efficiency of RNA interference. Transfection of cells with shOrai1 significantly downregulated the expression of Orai1 compared with transfection with shControl (Figure 4A). However, the reduction could be rescued by re-expression of Orai1 rescue construct (Figure 4A). Through Fluo-4–based intracellular Ca^2+^ measurement, we found that downregulation of Orai1 did reduce the amplitude of Ca^2+^ influx in tumor cells, but re-expression of the Orai1 construct could rescue the loss of Ca^2+^ influx (Figure 4B). Therefore, we obtained three groups of U251/SNB19 cells: the shControl group, the shOrai1 group, and the Orai1 rescue group.

To test our hypothesis, we detected the protein levels of total Pyk2 and p-Pyk2 in the three groups of U251/SNB19 cells. Western blot analysis showed that p-Pyk2 was significantly downregulated in shOrai1 cells, whereas total Pyk2 expression was comparable between the shOrai1 and shControl cells (Figure 4C). We also observed that Orai1 rescue was able to nearly counteract the decrease in p-Pyk2 caused by Orai1 downregulation (Figure 4C). The results suggest that Orai1 is essential for the phosphorylation of Pyk2. Meanwhile, when the total Pyk2 was knocked down by a Pyk2-specific shRNA plasmid (shPyk2) in the Orai1 rescue group, p-Pyk2 was also reduced (Figure 4C). Then, migration assays using Matrigel-coated Boyden chambers indicated that the number of migrating cells was significantly decreased in the shOrai1 group compared with the shControl group (Figure 4D). Similarly, re-expression of Orai1 could rescue the invasive ability of glioma cells (Figure 4D). However, the invasion of glioma cells in the Orai1 rescue group was suppressed again upon p-Pyk2 downregulation (Figure 4D).

### Orai1 regulated focal adhesion turnover and EMT-like via phosphorylation of Pyk2

**Figure 5:**
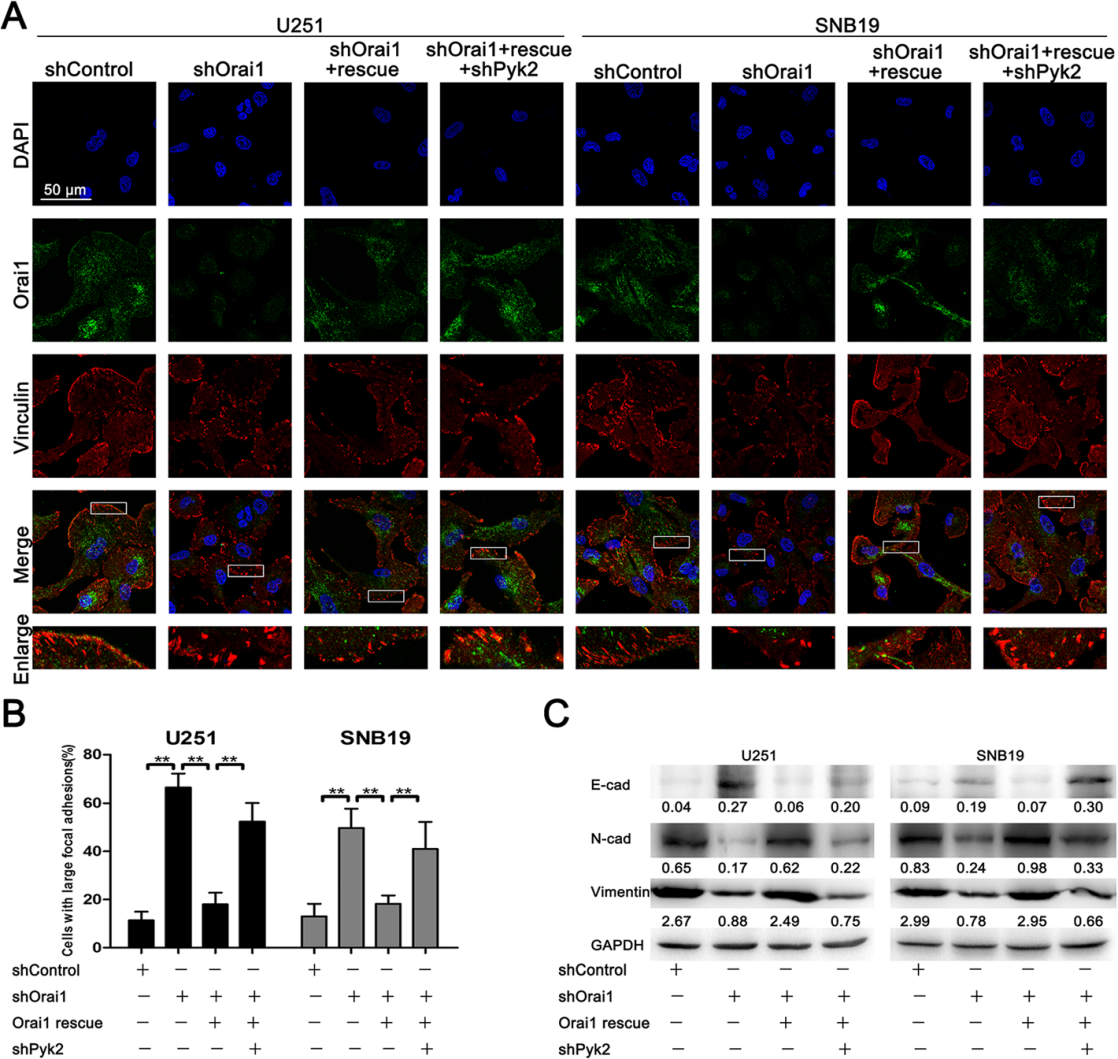
**Orai1 regulated focal adhesion turnover and EMT-like by modulating Pyk2 phosphorylation. (A)** Representative Orai1 (green) and vinculin (red) staining in U251 and SNB19 cells transfected with shControl, shOrai1, Orai1 rescue, or shPyk2. Magnification, ×1000. **(B)** Proportions of cells with large focal adhesions are presented as means ± SDs of nine different microscopic visions from three independent experiments. **P < 0.01. **(C)** Western blot analysis of E-cadherin, N-cadherin, and vimentin expression in U251 and SNB19 cells transfected with shControl, shOrai1, Orai1 rescue, or shPyk2.

To investigate the effect of Orai1 expression on focal adhesion turnover, U251/SNB19 cells transfected with shControl, shOrai1, Orai1 rescue, or shPyk2 were subjected to vinculin staining as described above. Similarly, we found that Orai1 downregulation led to cell rounding and induced large peripheral adhesions, although not as effectively as the inhibitor (Figure 5A). Re-expression of Orai1 rescued this change but Pyk2 downregulation increased the size of focal adhesions again (Figure 5A). Large focal adhesions were observed in 11%, 66%, 18%, and 52% of U251 cells and 13%, 50%, 18%, and 41% of SNB19 cells in the shControl, shOrai1, Orai1 rescue, and shPyk2 groups, respectively (Figure 5B).

We also postulated that Orai1 may influence the expression of EMT markers via the Pyk2 pathway. Western blot analysis showed that the expression of E-cadherin in the shOrai1 group was significantly increased, whereas the expression of N-cadherin and vimentin was significantly decreased compared to that in the shControl group (Figure 5C). We also observed that all three markers were restored to nearly the original levels in the Orai1 rescue group (Figure 5C). However, changes occurred again in the shPyk2 group (Figure 5C). These results suggest that Orai1 regulates EMT-like in glioma cells via the Pyk2 pathway.

## Discussion

In this study, we tested the hypothesis that the Ca^2+^ entry pathway SOCE is essential for glioma progression. This hypothesis was formed based on several previous reports that implicated a link between SOCE and a variety of tumors. The study of Yang et al. for the first time demonstrated the important role of SOCE in breast cancer progression [7]. They found that blocking SOCE impairs focal adhesion turnover, which can be rescued by the small GTPases Ras and Rac. Another study also showed that SOCE plays an important role in cervical cancer growth, migration, and angiogenesis [8]. However, the exact role of SOCE in glioma progression and its underlying mechanism have remained unclear. In the present study, we unraveled the role of SOCE in focal adhesion turnover and EMT-like in glioma cells, which involves modulation of Pyk2 phosphorylation. The major findings of this study are: (1) blockage of SOCE by a pharmacological inhibitor (SKF96365) or Orai1 downregulation can suppress glioma cell invasion and migration; (2) SKF96365 and Orai1 downregulation induce large focal adhesions and inhibit EMT-like in glioma cells; (3) SKF96365 and Orai1 downregulation reduce the phosphorylation of Pyk2; (4) re-expression of Orai1 can rescue all of the changes described above resulting from Orai1 downregulation; and (5) Pyk2 silencing inhibits cell invasion, induces large focal adhesions, and inhibits EMT-like in glioma cells again compared with the Orai1 rescue group.

The results of immunohistochemistry and western blot analyses indicated that the expression of Orai1, the key component of SOCE, is significantly correlated with the WHO grading of gliomas, with very low Orai1 expression in non-neoplastic brain tissues and very high Orai1 expression in glioblastoma samples and five glioma cell lines. Therefore, Orai1 may serve as a novel therapeutic target, and we selected Orai1 as the molecular target for studying the role of SOCE in subsequent experiments.

Two different approaches were employed to verify the role of SOCE in glioma cell migration and invasion. U251 and SNB19 cells were treated with the Ca^2+^ influx inhibitor SKF96365 and RNA interference, respectively. In the inhibitor group, we found that SKF96365 at the tested concentration (20 μM) did not impair cell viability but significantly inhibited the motility of glioma cells. Moreover, in the RNA interference group, we established glioma cell lines stably transfected with shControl, shOrai1, or Orai1 rescue. The results of Ca^2+^ measurements showed that Orai1 strongly controlled Ca^2+^ influx. Migration assays using Matrigel-coated Boyden chambers were performed to confirm the role of Orai1 in glioma cell invasion. The results showed that Orai1 promoted the invasive ability of glioma cells, suggesting that SOCE is crucial for the migration and invasion of glioma cells.

Several signaling molecules are believed to be regulated by Ca^2+^. Pyk2, also as known as cell-adhesion kinase β (CAKβ) or calcium-activation dependent tyrosine kinase (CADTK), is a new member of the focal adhesion kinase (FAK) family, with a highly homologous sequence to FAK [16]. Phosphorylation activation of Pyk2 via its Tyr402 residue usually depends on increasing intracellular Ca^2+^. An early study indicated that Pyk2 is located in focal adhesions and regulates multiple signaling events crucial for focal adhesion turnover [8]. Pyk2 is also known to be able to control cell motility through the regulation of genes associated with EMT [17,18]. Lipinski et al. demonstrated that Pyk2 plays a crucial role in the migratory behavior of glioblastomas [16,19]. Our study found that p-Pyk2 was expressed in different grades of glioma tissues, and increased with increasing malignancy of tumours (Additional file 1: Figure S1). However, despite this evidences for the role of Pyk2 in gliomas, the precise mechanism by which pyk2 promotes glioma dispersion remains elusive. Therefore, we tried to investigate the possibility that Pyk2 is an immediate effector of SOCE and that it controls the downstream mechanisms. The results from our study indicated that SOCE did regulate the phosphorylation of Pyk2. When SOCE was blocked by SKF96365, the expression of p-Pyk2 was decreased. Similarly, the phosphorylation of Pyk2 was under the control of Orai1.

Assembly and disassembly of focal adhesions are required for cell migration [20]. The continuous formation and disassembly of focal adhesions is termed focal adhesion turnover. The speed of focal adhesion turnover largely determines the speed of cell migration [7]. To investigate the mechanism by which SOCE controls glioma cell motility via the regulation of focal adhesion turnover, vinculin staining was employed to visualize focal adhesions. We found that SKF96365 induced large focal adhesions due to the resulting defects in focal adhesion turnover. Although not as effective as SKF96365, downregulation of Orai1 was able to create the same effect, whereas re-expression of Orai1 attenuated it. Then, to further understand the mechanism by which SOCE regulates focal adhesion turnover, we investigated the participation of Pyk2. When Orai1 rescue cells were transfected with a plasmid containing shRNA targeting Pyk2, large focal adhesions appeared again. A series of experiments demonstrated that SOCE regulates focal adhesion turnover via phosphorylation of Pyk2.

EMT, which is characterized by the loss of epithelial markers and the acquisition of mesenchymal markers, may enhance cancer cell migration and invasion in order to facilitate the development of metastasis [21-23]. There have been many thorough studies regarding EMT in cancers outside the CNS. However, the role of EMT in malignant gliomas still remains indistinct and controversial, likely because the brain lacks critical tissue components (i.e., epithelium and mesenchyme) [24]. The majority of GBMs do not show intrinsic E-cadherin expression [25], and only a small subfraction of highly E-cadherin–positive GBMs was observed [26]. Properly, EMT-like has been described to represent the EMT process in glioma [27]. In our attempt to test the function of SOCE in EMT-like, we examined the expression changes of three important EMT-related markers by western blot analysis. We found that control U251 and SNB19 cells almost did not express E-cadherin, whereas its expression was increased in SOCE-inhibited cells. N-cadherin and vimentin expression showed trends opposite to that of E-cadherin, suggesting that SOCE is crucial for EMT-like in glioma cells. Our results also demonstrate that Pyk2 participated in the regulation of EMT-like by SOCE.

## Conclusions

Our findings suggest that SOCE enhances glioma cell migration and invasion via regulation of focal adhesion turnover and induction of EMT-like. In addition, Pyk2 is an important immediate effector of SOCE that mediates downstream mechanisms. Thus, SOCE is likely to become a novel therapeutic approach in treating malignant glioma. Orai1, the key component of SOCE, may become a molecular target for glioma diagnosis and treatment.

## Additional file

Additional file 1: Figure S1. Expression of p-Pyk2 in human gliomas. **(A)** Immunohistochemistry of paraffin sections revealed that the expression levels of p-Pyk2 in non-neoplastic brain tissues and glioma tissues were quite different. NB, non-neoplastic brain tissues. Magnification, ×200. **(B)** Expression of p-Pyk2 in U251 and SNB19 glioma cells was detected by immunofluorescence. Magnification, ×400. **(C)** Western blot analysis of p-Pyk2 expression in non-neoplastic brain tissues, glioma samples (WHO II and IV), and two glioma cell lines.

## Abbreviation

SOCE: Store-operated Ca^2+^ entry
MTT: Methyl thiazolyl tetrazolium
CNS: Central nervous system
GBM: Glioblastoma multiforme
SOCs: Store-operated Ca^2+^ channels
STIM1: Stromal interacting molecule-1
ER: Endoplasmic reticulum
EMT-like: Epithelial-to-mesenchymal (−like) transition
DMEM: Dulbecco’s Modified Eagle’s Medium
FBS: Fetal bovine serum
WHO: World Health Organization
p-Pyk2: phosphorylated Pyk2
GAPDH: Glyceraldehyde 3-phosphate dehydrogenase
DMSO: Dimethyl sulfoxide
SDS: Sodium dodecyl sulfate
PAGE: Polyacrylamide gel electrophoresis
ECL: Enhanced chemiluminescence
CAKβ: Cell-adhesion kinase β
CADTK: Calcium-activation dependent tyrosine kinase
FAK: Focal adhesion kinase
