# Supplementary figures and images for: Store-operated Ca2+ entry regulates glioma cell migration and invasion via modulation of Pyk2 phosphorylation
Source: J Exp Clin Cancer Res. 2014 Nov 30;33(1):98. doi: 10.1186/s13046-014-0098-1 (PMC4258251; doi:10.1186/s13046-014-0098-1)

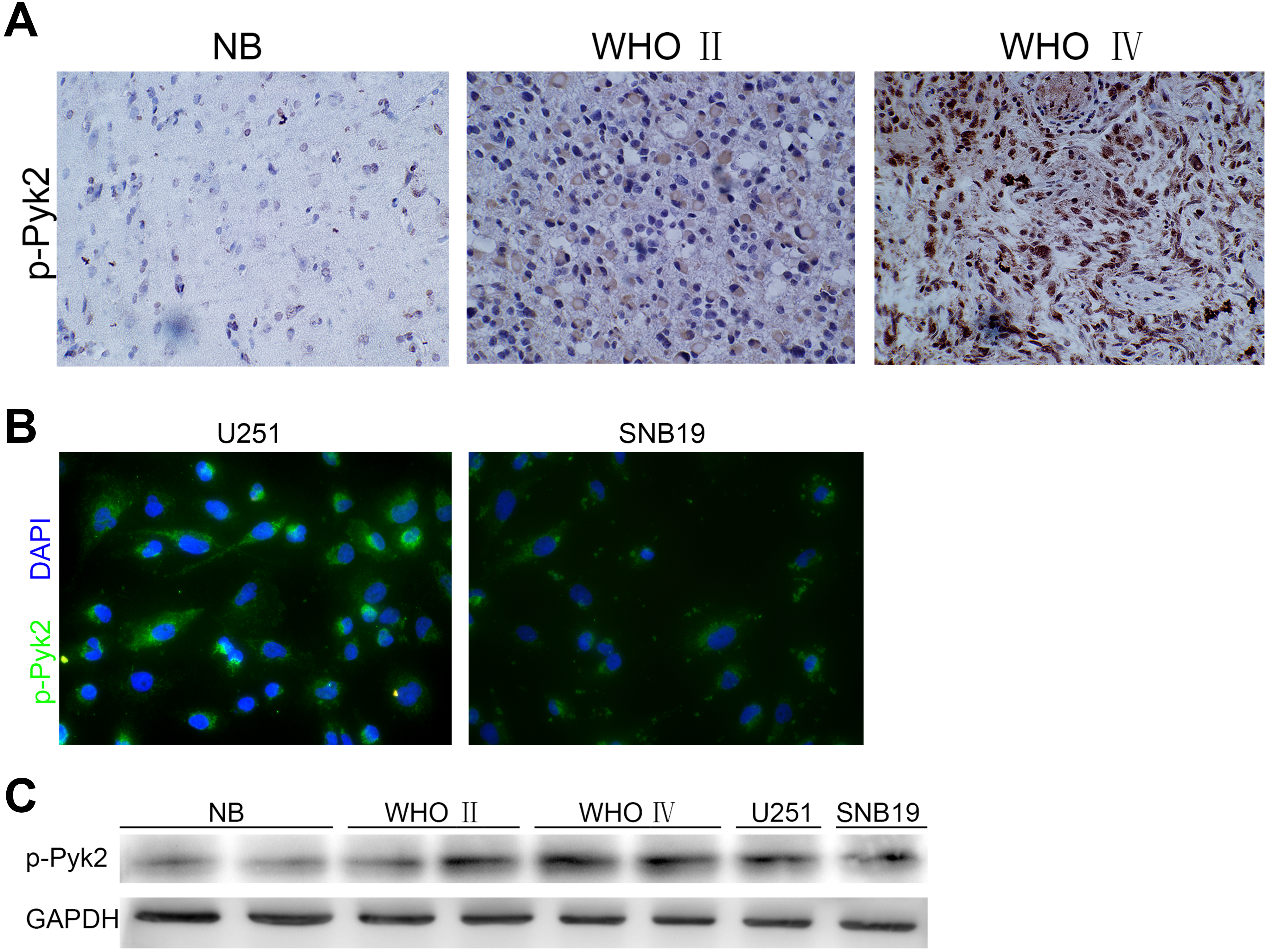

Supplement: Additional file 1: Figure S1. — Expression of p-Pyk2 in human gliomas. (A) Immunohistochemistry of paraffin sections revealed that the expression levels of p-Pyk2 in non-neoplastic brain tissues and glioma tissues were quite different. NB, non-neoplastic brain tissues. Magnification, ×200. (B) Expression of p-Pyk2 in U251 and SNB19 glioma cells was detected by immunofluorescence. Magnification, ×400. (C) Western blot analysis of p-Pyk2 expression in non-neoplastic brain tissues, glioma samples (WHO II and IV), and two glioma cell lines. [file 13046_2014_98_MOESM1_ESM.tiff]
